# Food Safety: Recommendations for Determining Doneness in Consumer Egg Dish Recipes and Measurement of Endpoint Temperatures When Recipes Are Followed

**DOI:** 10.3390/foods5030045

**Published:** 2016-06-23

**Authors:** Sandria Godwin, Curtis Maughan, Edgar Chambers

**Affiliations:** 1 Tennessee State University, 3500 John A. Merritt Boulevard, Nashville, TN 37209, USA; sgodwin@tnstate.edu; 2 Sensory Analysis Center, Kansas State University, Manhattan, KS 66502, USA; cmaughan@ksu.edu

**Keywords:** consumer food safety, egg dishes, temperature recommendations

## Abstract

Many consumers do not follow recommended food safety practices for cooking egg dishes, such as pies, quiches, and casseroles, potentially leading to foodborne illnesses such as Salmonellosis. The United States Department of Agriculture (USDA) recommends cooking egg mixtures until the center reaches 71 °C (160 °F). The objectives of this study were to determine what endpoint temperature information consumers receive from egg dish recipes, and if recipes would lead to safe temperatures when followed. Egg dish recipes (*n* = 226) from 65 websites, 50 cookbooks, and nine magazine titles (multiple issues of each) were analyzed. Time was the most frequently used indicator, given in 92% of the recipes, with 15% using only time. Other indicators included: set (89), browned (76), clean toothpick/knife (60), puffed (27), and jiggled (13). Only two recipes indicated final endpoint temperatures. Three recipes (a pie, a quiche, and an egg casserole) were chosen and prepared in triplicate to see if they would reach recommended temperatures. The pie and quiche were still liquid at 71 °C, and were well over the recommended temperature when cooked according to instructions, but the egg casserole was not consistently above 71 °C, when the recipe instructions indicated it was done and the center was light brown and “jiggled” This research indicates that consumers are not receiving information on endpoint temperatures in egg recipes, but the likelihood of foodborne illness is low since most dishes probably be cooked past the recommended temperature before the consumer considers them done unless there are many inclusions that may absorb liquid and reduce the appearance of liquid in the dish.

## 1. Introduction

Foodborne illness continues to be a public concern, with large social and economic tolls due to hospitalizations, loss of productivity, and death [1]. Many of these illnesses can be prevented through education and adherence to food safety recommendations, such as proper food handling, preparation, and storage [2]. One of the most common foodborne illnesses is *Salmonella*. Although there have been decreases in many other foodborne illnesses, *Salmonella* rates have remained steady [3]. The U.S. Food and Drug Administration (FDA) estimates around 142,000 illnesses each year are due to consumption of improperly prepared eggs that contain *Salmonella* [4], making eggs the principal risk factor for some strains of *Salmonella* [5,6]. European data suggests nearly 90,000 cases of foodborne illness related to *Salmonella* [7] and a British report specifically identifies undercooked egg dishes, eggs, and meat as the primary culprits [8].

Temperature recommendations for eggs when cooked by themselves vary in the US, with some guidelines stating only that they should be cooked until the yolk and white are firm, not runny [9] and others simply stating that all eggs should be cooked to 71 °C [10]. For dishes that include raw or undercooked eggs, such as ice cream, and salad dressing, it is recommended that pasteurized egg products be used [9,10]. Those same sources recommend that egg dishes such as casseroles be cooked until the internal temperature reaches 71 °C, although consumers have indicated that they do not use thermometers for egg dishes [11].

Common methods of egg cooking do not completely eliminate *Salmonella* in grossly contaminated eggs [12]. Although hard-cooking, soft-cooking, and poaching eggs have been shown to reduce *Salmonella* to a potentially safe level, other methods such as sunny-side-up, over-easy, and scrambling egg cooking do not adequately reduce bacteria levels [13,14]. Studies that have looked at *Salmonella* survival rates in eggs have typically been done with either whole or liquid eggs, and have not looked at more complicated dishes, such as casseroles and pies that might be the source of illness in the home [15].

One potential source of information on consumer food safety is recipes. Consumers have expressed that one of their preferred sources of food safety information was cookbooks and other print recipes [16]. A study by Maughan et al. showed that cookbooks with food safety information could alter the safety behaviors of consumers [17]. Unfortunately, food safety information has not been common in cookbooks [18].

The objective of this study was to determine what recommendations concerning determination of doneness consumers receive when using egg dish recipes found in cookbooks and internet sources, and if those recommendations are valid. A comparison of the information to the recommendations given by regulatory agencies, such as the FDA and USDA, will demonstrate gaps between what information consumers are being exposed to through recipes, and what information can potentially be added to recipes to improve food safety information for consumers. In addition, three recipes were selected from those surveyed and prepared in triplicate to determine if the recommendations given would lead to dishes that were safely cooked.

## 2. Study 1: Recipe Analysis

### 2.1. Methods

#### 2.1.1. Recipe Selection 

Recipes were selected from multiple sources used by consumers, both online and in print. Sources included retail locations (including supermarkets and kitchen supply stores that sell direct to consumers), blogs (both professional and personal, where recipes were the main focus), nonprofit (government, university extension sites, and organizations that promote eggs), magazines, cooking shows, traditional cookbooks, and local cookbooks (from local community organizations in Nashville, TN). The number and types of recipes used from each source are shown in Table 1.

In total, 226 egg recipes were analyzed for this study. Recipes were chosen where eggs were one of the critical components, and where they could potentially be undercooked. Both those that used the whole egg and those that used either the yolk or whites only were included in the analysis, though the focus was predominantly on whole eggs. These recipes came from 65 websites, 50 cookbooks, and nine magazine titles (with multiple issues of each). Recipe types included for analysis were (in decreasing order of frequency): pie, quiche, casserole, frittata, custard, strata, soufflé, omelet, cheesecake, pudding, torte, and other.

Pies included in these recipes were mostly custard and cream type pies, such as pumpkin, lemon, custard, and coconut. When recipes fell into two categories, such as a custard put into a pie, the category where the final cooking of the eggs happened was selected. Recipes that were unique and did not fall into any category were placed in the “other” category.

#### 2.1.2. Recipe Analysis

The directions of each recipe were categorized by what method was recommended to the consumer for determining when the dish was finished cooking. The majority of the recipes gave more than one method for determining the doneness of the product, so an overall count of indicator type was used. Where recipes used different wording to describe the same method, they were combined into a single category; for instance, “browned,” “golden brown,” and “light brown” were all combined into one category, as were the variations of utensils, including using a fork, knife, or toothpick. Other directions that were only seen once, and therefore not placed in a category, included instructions such as “cooked through,” “edges pull away,” and “clumped.” Categorization of the recipes was done by two separate researchers, and then compared for accuracy and consistency. Any differences were resolved by a third researcher.

### 2.2. Results

#### 2.2.1. Recipe analysis

Recipes gave one (22%), two (46%), three (27%), or four (5%) indicators of doneness. Only two recipes gave no indicator of doneness. Of those recipes that gave only one indicator, the most common (78%) was time. 

Indicators used by the recipes in order of frequency included cooking time (92%), being “set” (39%), brownness (34%) , having a probe or utensil come out clean (27%), puffiness (12%), reduced jiggling (6%), other (5%), thickening (3%), bubbling (3%), final temperature (1%), and raw eggs (1 recipe). Time (or a range of time) was by far the most common indicator given, found in 92% of the recipes. Those recipes that gave no other method of determining doneness other than time could be especially difficult for consumers who were not familiar with preparing the product and knowing when to stop cooking the items. This could result in undercooked items that carry an increased risk of leading to foodborne illness.

Approximately 40% of recipes used the word “set” as a doneness indicator with over half (54%) stating only “until set,” and others specifying what part of the dish should be set, whether sides (27%), top (9%), center (6%), or bottom (4%). The bottom being set was used in recipes such as frittatas where the eggs were undisturbed during the cooking process. It is important to note that those that gave a location other the center could have resulted in a center that was not set or not cooked completely.

Only two of the recipes, both from retail sources, gave an endpoint temperature for the egg dish and both recommendations met USDA minimum temperature guidelines. One, a strata with ham, recommended that the thermometer inserted in the center read 170 °F (76.7 °C), while the other, a custard pie, recommended 160 °F (71 °C) as an endpoint temperature for the custard before baking the pie. Both still used time as the primary method for determining doneness, with temperature as a secondary means of checking the product if a thermometer was used.

#### 2.2.2. Discussion

Almost all of the indicators of doneness found in these recipes were based on methods that were either visual (color, set, or a probe coming out clean) or time based. Almost none of the recipes gave a temperature to reference during the cooking procedure. This shows that consumers are not receiving the information on endpoint temperatures recommended by the USDA in the recipes that they likely use for preparing egg dishes.

Based on the findings of this study that almost no recipes contain temperature information, a second study was conducted to determine if the directions given would give lead to undercooked egg dishes that could increase the risk of foodborne illness. The temperature recommendations given by USDA and other groups are based on reducing the risk of foodborne illness, rather than on consumer measures such as doneness of the dish [19]. It was unknown whether following the instructions in consumer recipes for egg dishes would lead to dishes that were at or above the recommended 71 °C.

## 3. Study 2: Testing Recipe Instructions

### 3.1. Materials and Methods

Three recipes were chosen for testing the instructions, a lemon chess pie, a quiche, and an egg based casserole. Recipes were chosen to represent different types of recipes with varying difficulty and inclusions. The chess pie was the simplest with no inclusions, the quiche had some inclusions as well as cheese which could change the texture and browning appearance, and the casserole had large inclusions including chunks of bread. Ingredients for each recipe were purchased at a local supermarket. A test item from each recipe was baked to test proper placement of instrumentation for each phase of the experiment, and recipe measurements were modified slightly for consistency. Household measures were changed to standard metric weights and volumes for cooking. Where applicable, standard sizes were obtained from the National Nutrient Database-Foods list [20]. For example, mixed, pooled eggs were added in 50 g increments based on the listed weight of a large egg in the database. A laboratory balancing scale (model SP2001, OHAUS, Parsippany, NJ, USA) was used to measure ingredients. A 250 mL graduated cylinder was used to measure liquid ingredients. Observations and recordings were conducted on three replications of each recipe. 

Oven temperature was monitored using a calibrated oven thermometer (Farberware, Garden City, NY, USA). An oven safe cooking thermometer and a digital thermometer with probe (Taylor, Oak Brook, IL, USA) that had been previously checked for consistency and calibrated for accuracy were used to monitor the temperature of the dishes. Thermometers were affixed to a ring stand with clamps for support and stability, and to ensure that the thermometer placement and depth were consistent. Thermometers were placed in the center of the dishes at a 45° angle, not touching the bottom of the pan or the crust. When a dish reached the recommended safe temperature (71 °C) the cooking time that had passed was noted, and a spoon was placed into the center of the dish to check for consistency. The dish was then allowed to continue the cooking process until the time stated on the recipe, at which point it was removed from the oven, placed on a wire cooling rack, and temperature readings were taken both immediately and after the item was allowed to rest before cutting, to mimic typical consumer behavior as listed in the recipes or in similar recipes (2 h for the pie, and 10 min for the quiche and casserole). 

Subjective measurements of set, coloring, jiggling, clean toothpick, and clean knife tests were assessed by two observers and recorded by video and photography. An additional factor of “puff” was assessed in the quiche recipe due to doneness instructions. Definitions for the measurements were agreed upon before assessment began. Consistency was assessed at the time that the item reached 71 °C by dipping a spoon into the center of the item, and was defined as the holding together or retention of form, and was assessed as to being liquid, semi-formed, or firm. All other evaluations were done immediately upon removal from the oven once the cooking time was complete, with the exception of the clean slicing, which was done after the resting period. 

Set was determined to mean if the item had a fixed or rigid appearing center. Jiggle was used to describe whether an item moved lightly and quickly from side to side or up and down when given a quick jerk, i.e., like gelatin. Color change was noted as no change, golden, light brown, or brown when assessing the outer top of the finished product. The clean toothpick and knife tests were defined as the amount of adherents on the instrument after it was inserted into the center of the item and removed.

### 3.2. Recipe Preparation

#### 3.2.1. Recipe 1—Lemon Chess Pie

The first recipe tested was a lemon chess pie, modified as follows, with the SI measurements in parenthesis [21].

- 2 cups sugar (454 g)
- 1 tablespoon all-purpose flour (7 g)
- 1 tablespoon cornmeal (7 g)
- ¼ teaspoon salt (0.2 g)
- ¼ cup butter or margarine, melted (57 g)
- 2 teaspoons grated lemon rind (15 g)
- ¼ cup lemon juice, fresh (60 mL)
- ¼ cup whole milk (60 mL)
- 4 large eggs, grade A (200 g)
- 1 unbaked 9-inch pastry shell, frozen

**Preparation:** Combine sugar, flour, cornmeal, and salt. Add butter, lemon rind, lemon juice, and milk: mix well. Add eggs, one at a time, beating well with a wire whisk after each addition. Pour into pastry shell. Bake at 350 °F (177 °C) for 50 min. Yield: one 9-inch pie

The uncooked pie crust was set out on the counter (between 23 °C and 26 °C) to defrost 1 h before cooking time along with the butter, which was allowed to soften before mixing. Dry ingredients were weighed and stirred together before wet ingredients were added to the mixing bowl. Eggs were combined, beaten, and then added to the mixture in 50 g increments, with beating in between each addition. The mixture was poured into the pie crust and placed in a preheated Kenmore 24 inch oven, with a timer set for 50 min. Time and consistency were noted once the dish reached 71 °C, and then it was allowed to continue cooking until the time stated in the recipe.

After the 50 min of cooking time was completed and temperature and consistency measures were noted, the pie was placed on a wire rack and allowed to cool for 2 h, at which point the internal temperature was again taken and recorded.

#### 3.2.2. Recipe 2—Quiche

The quiche recipe was adapted as follows, with standardized measurements in parenthesis [22].

- 1 unbaked deep dish 9-inch pastry shell, frozen
- 3 cups shredded cheddar cheese (170 g)
- 4 eggs (200 g)
- 1 cup whole milk (236 mL)
- 1/2 cup heavy cream (118 mL)
- 3 tablespoons flour (19 g)
- 1–2 teaspoons dried chives (0.1 g)
- 1/4 teaspoon salt (1.2 g)
- 1/8 teaspoon pepper (0.2 g)

Preparation: Preheat oven to 375 °F (191 °C). Let frozen crust stand at room temperature for 1 h to thaw. Sprinkle cheese into the bottom of the pie crust; set aside. In medium bowl, combine remaining ingredients and beat with a wire whisk until smooth and incorporated. Pour carefully over cheeses in crust. Bake for 40–50 min until quiche is puffed, golden brown, and set. Let stand 5 min, cut into wedges to serve.

The pie crust was set out on the counter (23 to 26 °C) 1 h before cooking time to thaw. Dry ingredients were weighed and mixed together, followed by wet ingredients as stated in the recipe. A wire egg whisk was used to beat ingredients thoroughly. The mixture was then poured over the cheese-filled pie crust and placed in the preheated oven. The temperature of the quiche was monitored for an internal temperature of 71 °C, at which point the time was recorded and the consistency of the quiche was checked by placing a spoon into the center of the quiche.

The item was allowed to continue cooking for the set 40 min and the temperature was taken, at which point it was allowed to cook the entire 50 min stated in the recipe. The quiche was then removed from the oven and placed on a wire rack on the counter. Final temperatures were taken and the quiche was assessed for consistency, set, color, jiggling, and whether a toothpick and knife came out clean. Temperatures were recorded from the center of the dish, but various spots around the quiche were checked to ensure uniformed heating. The quiche was allowed to set for 10 min and again assessed for temperature and sliced.

#### 3.2.3. Recipe 3—Breakfast Casserole

The breakfast casserole was adapted as follows, with standardized measurements in parenthesis [23].

- 1 pound sausage (454 g)
- 9 eggs (450 g)
- 1.5 cups whole milk (355 mL)
- 1 cup grated cheese (113 g)
- 1 teaspoon salt (4.8 g)
- 1/2 teaspoon pepper (1.1 g)
- 1 teaspoon dry mustard (2 g)
- 4 slices bread, cubed (94 g)

Preparation: Brown sausage; drain. Mix eggs, milk, salt, pepper, and mustard together. Add meat and cheese. Mix well and pour over bread; toss lightly. Pour into buttered baking dish. Refrigerate overnight. Bake at 350 °F (177 °C) for 45 min. Let stand for about 10 min before serving.

The sausage was fried and drained, and had a final resting weight of 219 g on average. Other ingredients were mixed and added together as instructed. An 8″ × 8″ pan was buttered with a 5 g pat of butter prior to adding the mixture. Plastic wrap was placed over the dish, which was refrigerated overnight for 14 h. The casserole was then removed from the refrigerator and allowed to wait while the oven preheated, and then was placed in the oven with a thermometer inserted into the center and the timer set for 45 min. The casserole was checked at 45 min, at which point none of the repetitions had reached the recommended temperature and were still in a semi-liquid state, and so were allowed to continue cooking. When the product reached 71 °C, a second thermometer was used to check the internal endpoint temperature, and consistency was checked. The dish was then removed from the oven and placed on a wire rack to cool. The casserole was then checked for set, color, jiggling, and the clean knife and toothpick test. Final temperatures were taken after 10 min, and the casserole was then sliced and plated.

### 3.3. Results

Table 2 shows the results of both cooking trials. The lemon pies reached 71 °C at an average of 28 min and were all still liquid in consistency. After the recommended cooking time of 50 min had passed, the pies had reached an average temperature of 92 °C and were all were considered set in consistency. One of the pies (Pie 1) took longer to reach 71 °C than the other two pies and did not brown by the end of the cooking period despite a similar endpoint temperature to Pie 3. It is unclear why there was a difference in browning. This could have been due to variances in the oven, although oven temperature was checked before cooking each item. One of the pies (Pie 2) had a slight jiggle in the center, and all had a clean toothpick and a clean knife test. The average temperature of the pies after they had been allowed to rest for 2 h on a wire rack was 36 °C. At that time the pies were sliced and varied between a semi clean slice and a clean slice when plated.

The quiches reached 71 °C at an average of 34 min and were all still liquid in consistency. They reached an average temperature of 79 °C at 40 min and 81 °C at 15 min. The quiches were fairly consistent as finished products. They were all set, did not puff or jiggle, and had clean knife and toothpick tests. All of the quiches had a light brown top coloring and reached a resting temperature of 79 °C at 10 min. When plated, all were semi clean slices.

The casserole had the only problematic results of any of the dishes. At the cooking time given in the recipe (45 min), all of the replicate dishes were still under the recommended temperature, and all of them had some liquid remaining in the center and on the top of the dish and did not look done. Would a consumer use the time as the definite endpoint or cook longer to get a casserole that was set and not runny? Once the casseroles reached 71 °C, all of them were considered set, though some still had some jiggle. After a 10 min resting period, the average temperature of the casseroles was 67 °C, and all of them sliced cleanly.

### 3.4. Discussion

Limitations to this study included the limited number of recipes that were prepared and observed. Also, this study did not attempt to inoculate recipe preparations and measure bacterial loads to determine what level of *Salmonella* would be destroyed through the cooking process. Some studies have shown that the ability of *Salmonella* to survive cooking in eggs can be dependent on factors such as pH, heat resistance, initial bacterial load, and previous exposure to environmental stress [24]. For this reason, consumers should only obtain eggs from approved sources and refrigerate them until use, in addition to cooking them thoroughly. This study assumes proper handling of eggs has occurred before cooking, and gross contamination has been avoided.

In all of the trials of the pies and quiches, the products achieved the target temperature of 71 °C well before the indicated cooking time had been reached. Upon reaching 71 °C, considered to be safe in terms of reduced risk for harboring a foodborne pathogen, recipes were still in a liquid phase and typically would not have been eaten or considered finished by consumers. Once the products were cooked for the recommended time in the recipes, they were all well above the recommended safe temperature, and all had a final set consistency, clean toothpick, and clean knife test.

The breakfast casseroles was unique in that the replications took longer than the recommended time to reach the appropriate temperature. The observers also noted that it was more difficult to determine if the products were “set” compared to other dishes due to not being able to determine if the liquid on top was from uncooked eggs or from oils from the cheese or sausage. At the 45 min mark, there was some light browning despite the liquid on top, meaning some consumers may have chosen to remove the dish at that point, though it is likely that those familiar with the type of dish would have continued cooking it, especially given that the dish still jiggled markedly and appeared undercooked.

One of the main reasons for the recommendation of cooking egg dishes to a temperature of 71 °C is to ensure thermal inactivation of bacteria that cause foodborne illness such as strains of *Salmonella*. The thermal death rate of these bacteria can depend on multiple factors, including pH and additives such as salt. Some studies have shown that the thermal death rates of *Salmonella* spp. Do not always follow a log-linear curve, making it difficult to predict when a product has reached a specific death rate [25]. From research using whole eggs, we can estimate that from the minimum temperatures reached after 45 min (61 °C), the product should only have needed to remain at that temperature for a little over a min to be safe (assuming a 7-D reduction needed when *D_60_* = 0.17 min [26]). Although this is an approximation, it can reasonably be assumed that most consumers would be safe at that point, though cooking to a higher temperature would be preferred.

The results of these trials indicate that consumers are at minimal risk for undercooking simple egg dishes such as pies and quiches, since they would continue cooking them past the liquid stage that was seen at 71 °C, though some caution should still be exercised with more complex egg dishes such as casseroles.

## 4. Conclusions

Efforts to educate consumers on egg safety should prioritize the safe handling of raw eggs and the reduction of cross contamination, rather than endpoint temperatures. Almost no recipes contain endpoint temperatures for egg dishes, despite the recommendations of various food safety organizations. Although this does seem like a potential concern in theory, the risk appears to be minimal in practice based on our results. We found that simple egg dishes did not solidify until well after they reached the recommended temperature of 71 °C, at which point the risk for foodborne illness would be minimal. Consumers would likely also reach a high enough temperature for thermal death of *Salmonella* spp. in more complex dishes such as casseroles as well, though there may be some risk based on their interpretation of when a dish is considered set. Following the directions found in egg dish recipes led to safely cooked dishes in terms of endpoint temperature, with the possible exception of the casserole. Microbial testing for more complex egg dishes may need to be done to ensure that they are indeed safe based on the given directions, and additional trials of other egg dishes could be done to determine if this applies to other egg products as well. Food safety agencies and educators can use this information to focus on other products or aspects besides egg dish temperature where there is a greater risk for foodborne illness.

## Figures and Tables

**Table 1:** Sources of recipes used in the study, along with types of recipes from each source.

Source	Type	No. of Recipes	Pie	Quiche	Casserole	Frittata	Custard	Strata	Soufflé	Omelet	Cheesecake	Pudding	Torte	Other
Internet	Retail	24	12	11	0	0	0	1	0	0	0	0	0	0
	Nonprofit	14	3	7	2	2	0	0	0	0	0	0	0	0
	Blogs	13	3	7	1	1	0	0	1	0	0	0	0	0
	Magazines	10	3	4	1	0	0	2	0	0	0	0	0	0
	Cooking Shows	9	1	1	0	1	1	2	0	0	0	1	2	0
Print	Traditional Cookbooks	72	18	13	4	10	9	3	6	5	1	1	0	2
	Local Cookbooks	59	26	3	13	2	4	1	2	0	4	2	1	1
	Magazines	25	8	4	3	4	1	1	0	0	0	0	0	4
TOTAL		*226*	*74*	*50*	*24*	*20*	*15*	*10*	*9*	*5*	*5*	*4*	*3*	*7*

**Table 2:** Temperature and subjective measurements of the egg dishes during and after the cooking process.

Item	Time to 71 °C	Consist. at 71 °C	Temp. at Time in Recipe	Jiggle	Color	Toothpick	Knife
Pie 1	36 min	Liquid	89 °C	None	No change	Clean	Clean
Pie 2	24 min	Liquid	98 °C	Jiggle	Brown	Clean	Clean
Pie 3	24 min	Liquid	90 °C	None	Brown	Clean	Clean
Quiche 1	35 min	Liquid	79 °C	None	Lt brown	Clean	Clean
Quiche 2	33 min	Liquid	79 °C	None	Lt brown	Clean	Clean
Quiche 3	35 min	Liquid	78 °C	None	Lt brown	Clean	Clean
Casserole 1	59 min	Set	65 °C	Jiggle	Lt brown	Clean	Clean
Casserole 2	61 min	Set	66 °C	Jiggle	Lt brown	Clean	Clean
Casserole 3	54 min	Set	61 °C	None	Lt brown	Clean	Clean
